# Motif prediction to distinguish LPS-stimulated pro-inflammatory *vs*. antibacterial macrophage genes

**DOI:** 10.1186/1745-7580-6-5

**Published:** 2010-09-21

**Authors:** Rahul K Kollipara, Narayanan B Perumal

**Affiliations:** 1 School of Informatics, Indiana University-Purdue University Indianapolis, Indianapolis, Indiana 46202, USA

## Abstract

**Background:**

Innate immunity is the first line of defence offered by host cells to infections. Macrophage cells involved in innate immunity are stimulated by lipopolysaccharide (LPS), found on bacterial cell surface, to express a complex array of gene products. Persistent LPS stimulation makes a macrophage tolerant to LPS with down regulation of inflammatory genes ("pro-inflammatory") while continually expressing genes to fight the bacterial infection ("antibacterial"). Interactions of transcription factors (TF) at their cognate TF binding sites (TFBS) on the expressed genes are important in transcriptional regulatory networks that control these pro-inflammatory and antibacterial expression paradigms involved in LPS stimulation.

**Results:**

We used differential expression patterns in a public domain microarray data set from LPS-stimulated macrophages to identify 228 pro-inflammatory and 18 antibacterial genes. Employing three different motif search tools, we predicted respectively four and one statistically significant TF-TFBS interactions from the pro-inflammatory and antibacterial gene sets. The biological literature was utilized to identify target genes for the four pro-inflammatory profile TFs predicted from the three tools, and 18 of these target genes were observed to follow the pro-inflammatory expression pattern in the original microarray data.

**Conclusions:**

Our analysis distinguished pro-inflammatory *vs*. antibacterial transcriptomic signatures that classified their respective gene expression patterns and the corresponding TF-TFBS interactions in LPS-stimulated macrophages. By doing so, this study has attempted to characterize the temporal differences in gene expression associated with LPS tolerance, a major immune phenomenon implicated in various pathological disorders.

## Background

Innate immunity, one of the two arms of the immune system, provides the first line of defence against pathogens in mammals and nearly all other living things. Animals in the lower evolutionary scale, such as insects, fight off infections solely employing innate immune mechanisms. The innate immune system quickly alerts the host of the presence of microbial pathogens and this response is mediated through the expression of a limited number of receptors called pattern recognition receptors (PRRs) to identify pathogen associated molecular patterns (PAMPs) expressed by invading pathogens. Infections trigger PRRs, such as toll-like receptor (TLR) genes [1], to recognize PAMPs of invading pathogens and prompt an intracellular signalling cascade which ends in induction of pro-inflammatory cytokines, chemokines, type I interferons, and antimicrobial effectors that are essential for providing continuous protection from infection. In highly evolved organisms (starting from jawed vertebrates) innate immunity is required for priming adaptive immunity, the second arm of the immune system with long-term and specific immune response [2]. Macrophages are immune cells highly involved in conducting innate immune responsibilities and also play a role as an antigen-presenting cell (APC) to T lymphocytes (in adaptive immunity); they express various TLR genes to mediate the pro-inflammatory and antimicrobial responses.

LPS, a major component of gram-negative bacterial cell surface, is a potent stimulator of macrophages. LPS acts *via *the TLR4 receptor to trigger downstream signalling and expression of pro-inflammatory and antibacterial genes [1]. This induction needs to be under tight control since dysregulated inflammation can cause a number of pathological disorders such as septic shock, autoimmunity, atherosclerosis and cancer [3]. Various mechanisms of negative regulation of TLR-induced gene expression have been proposed to dampen uncontrollable inflammation [4] and these collectively lead to the phenomenon of "LPS tolerance" [5] wherein there is decreased expression of pro-inflammatory genes when there is prolonged LPS administration. Foster *et al *[6] have characterized the gene expression profiles of macrophages differentially treated with LPS to classify the genes into various phenotypic states including a tolerant state obtained by an initial LPS treatment. Their analysis of the genes expressed in the tolerant phenotype categorized the genes as belonging to "tolerizable" or "non-tolerizable" sets depending on no induction or further induction respectively during a second LPS treatment compared to the first one. Although LPS tolerance could prevent pathological inflammatory conditions in chronic bacterial infections, there is a strong need for a persistent antibacterial response to keep the infections under control. The set of genes that exhibit the tolerizable phenotype can be considered "pro-inflammatory" while those belonging to the non-tolerizable phenotype as "anti-bacterial".

Transcriptional regulation is a crucial biological mechanism controlling gene regulation in the tolerant phenotype *vs*. the basal state that corresponds to no LPS stimulation. A number of studies have looked at the transcriptional programs in LPS-mediated macrophage stimulation including the roles of TFs in prolonged LPS treatment [4,7-9]. Roach et al [7] carried out a holistic approach to identify 92 TFs in human macrophages stimulated with LPS; however, this study did not distinguish between the tolerant and basal states since there was no re-stimulation with LPS in this study. A recent study characterized the Cebpd TF as a potential regulator of a switch between the basal and tolerant state [9]. A microarray analysis performed by Mages et al [10] similar to the Foster et al [6] experiment employed in our study, observed diminished gene expression of a vast majority of LPS-induced genes upon a second LPS treatment (tolerizable). However, these authors did not characterize any of the TF-target interactions responsible for the various phenotypes distinguished in their analysis. In order to achieve a global perspective on the transcriptional regulatory mechanisms inherent in LPS tolerance, we examined the Foster *et al *microarray data [6] for the control of pro-inflammatory *vs*. antibacterial gene expression. Using bioinformatics approaches, we characterized TF-TFBS interactions that differentiate LPS-stimulated pro-inflammatory *vs*. antibacterial gene expression depending on the well-accepted premise of coordinate expression corresponding to appropriate TF-TFBS interactions. Further, we show that some of these TF-TFBS interactions predicted from our analysis have been biologically validated as transcriptional targets in the literature with evidence of roles in LPS tolerance.

## Results

### Classification of antibacterial and pro-inflammatory macrophage genes

**Figure 1:**
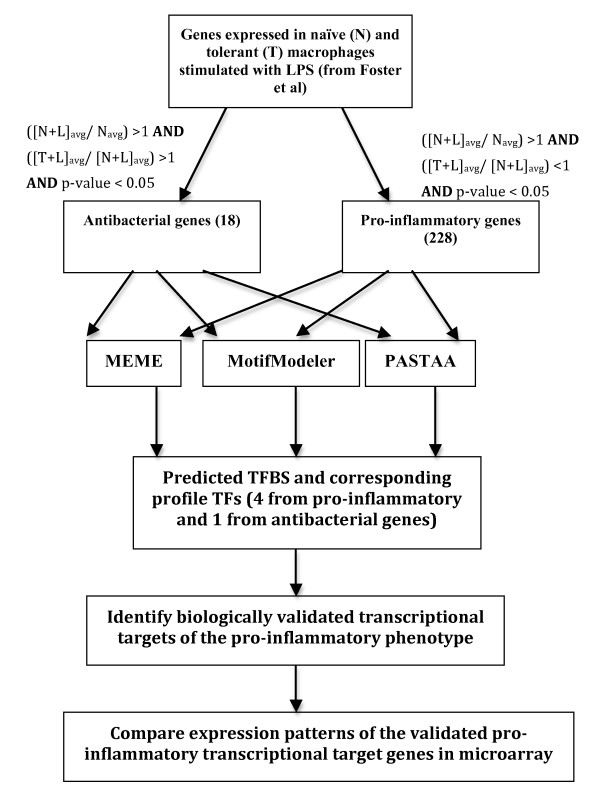
**Workflow of experimental analysis to distinguish transcriptomic signatures of pro-inflammatory *vs*. antibacterial genes**. Average gene expression values in untreated macrophages, (N)_avg_, 4 hrs LPS-treated macrophages, (N+L)_avg_, and 24 hrs LPS-treated followed by 4 hrs re-treated macrophages, (T+L)_avg _were calculated. Two sets of genes were classified by the conditions mentioned in Methods.

**Figure 2:**
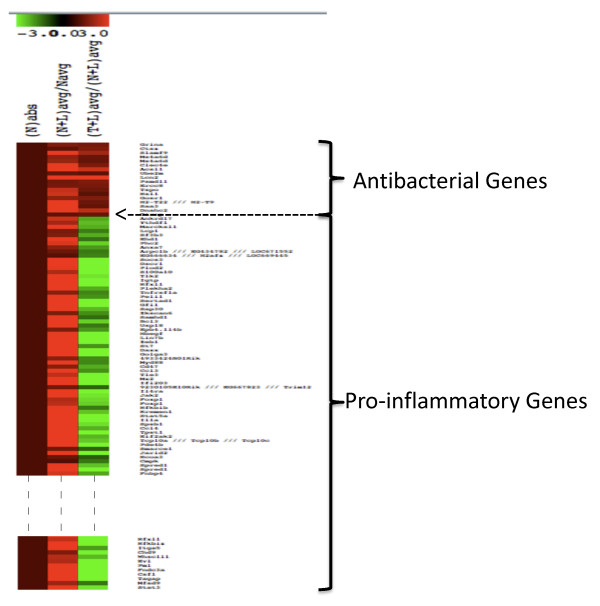
**Heat map showing the differential expression patterns of LPS-stimulated genes**. N_avg_, (N+L) _avg _and (T+L) _avg _are as described in Figure 1. N_abs _was introduced to distinguish the patterns clearly and it is equal to one. The rationale was that the fold change is >1 for both classes of genes in the N+L phenotype, and <1 for pro-inflammatory and >1 for the antibacterial class genes in the T+L phenotype. Only a subset of the pro-inflammatory genes is shown.

In order to distinguish macrophage genes responsible for antibacterial *versus *pro-inflammatory phenotypes upon induction of LPS tolerance, we filtered the differentially expressed genes reported in Foster *et al *[6] as shown in the top part of Figure 1. These authors have performed two replicates of microarray hybridizations for each of the following experimental conditions: The naïve (N) state that corresponds to no LPS treatment, a 4-hour treatment of LPS to naïve cells (labelled N+L) and a 24-hour LPS treatment of naïve cells followed by an additional 4-hour treatment (labelled T+L). Upon the first LPS treatment for 24 hours, the macrophages in the naïve state become tolerant and a second treatment for an additional 4 hours should reduce inflammatory gene expression but sustain or increase antibacterial gene expression. We employed a novel filtering approach to distinguish the Foster *et al *microarray genes into the pro-inflammatory and antibacterial classes. Essentially if a gene's expression level is X in N, for it to be a pro-inflammatory gene its expression in N+L, Y should be greater than X (Y > X) but expression in T+L, Z should be less than Y (Z < Y). On the other hand if a gene is expressed higher in N+L compared to N (*i.e*., Y > X) and is same or increased in T+L compared to N+L, (*i.e*., Z≥Y although we filtered only when Z > Y), then it was classified as antibacterial. This filtering paradigm is based on the rationale that while pro-inflammatory genes are down regulated in the tolerant state, antibacterial genes are required for infection control. We obtained 228 pro-inflammatory and 18 antibacterial genes (p < 0.05 significance) from an annotated set of 45,101 probes in the microarray indicating the stringency in our phenotypic restrictions (Additional file 1, Table S1). This categorization is further confirmed when we clustered the original gene expression data of a mixture of these genes (228 + 18 = 246) using the MeV software [11]. As expected, the two classes of genes clearly separated into two clusters (Figure 2) with the pro-inflammatory set showing down regulation and antibacterial set showing up-regulation compared to the tolerant state (T+L/N+L avg).

### Gene ontology (GO) analysis of the two gene sets

**Figure 3:**
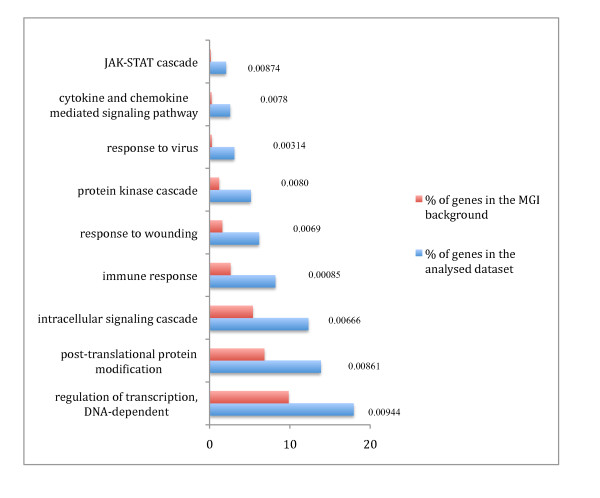
**GO analysis of the pro-inflammatory genes**. GO analysis of the pro-inflammatory genes based on annotated biological process GO terms was done using GOstat [37]. Enrichment of the pro-inflammatory gene set compared to a Mouse Genome Informatics (MGI) murine genome background d for each GO term is shown with significant p-values (< 0.01).

The pro-inflammatory genes could be classified into 13 categories of GO terms while the smaller antibacterial gene set mapped only to two GO categories. Figure 3 shows a subset of GO terms from the pro-inflammatory gene set showing significant enrichment (p < 0.01) in a number of biologically relevant categories such as immune response, cytokine and chemokine mediated signalling pathway, and DNA-dependent regulation of transcription.

### Characterization of TFBS in the two data sets

**Figure 4:**
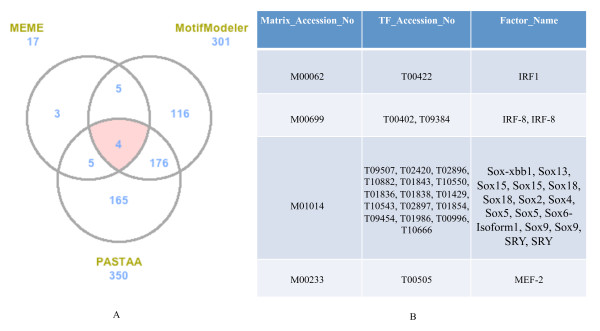
**Comparison of predicted TFBS from MEME, MotifModeler, and PASTAA**. A. Venn diagram showing numbers of predicted motifs from the three tools for the pro-inflammatory genes. The numbers next to the prediction tool names are the total numbers of top 70% predicted TFBS for each tool. B. List of common predicted motifs and the corresponding profile TFs. Profile TFs corresponding to these four common TFBS were obtained from TRANSFAC [15].

In order to correlate gene expression with transcriptional regulation, we set out to identify characteristic TF-TFBS interactions unique to the two classes of genes. We approached this by employing three independent TFBS motif detection tools with different algorithmic paradigms and to arrive at a list of predicted TFBS common to the three tools *viz*., MEME, MotifModeler and PASTAA (Middle part of Figure 1). MEME is an expectation-maximization tool that fits a two-component finite mixture model to the input sequences for motif prediction [12]. MotifModeler uses a model selection approach that best fits a set of motifs to gene expression values (both up and down regulated) in co-ordinately expressed genes [13]. PASTAA detects TFBS based on the prediction of binding affinities of a TF to promoters and their association with tissue specific expression of corresponding genes [14]. To reduce false positives, we considered only the top 70% predicted TFBS from each tool. MEME, MotifModeler and PASTAA individually identified 17, 301 and 350 motifs respectively from the pro-inflammatory genes while four motifs were found to be common to all three tools (Figure 4A). Figure 4B lists the TFBS common to all tools found in the pro-inflammatory gene set and the corresponding profile TFs from TRANSFAC [15]. We compared scores from the three TFBS prediction tools for the four motifs identified in our pro-inflammatory data set, and similarly predicted from a random set of 228 genes. As shown in Table 1, the scores of MotifModeler and PASTAA from the pro-inflammatory gene set are significantly higher than the scores from the random gene data indicating genuine enrichment of these motifs in our genes with a potential for transcriptional control of pro-inflammatory specific gene expression. MEME did not predict any of these four motifs in the random gene set. The two motifs predicted from PASTAA and MotifModeler when a similar analysis as described above was performed on the random gene set showed reciprocal higher (PASTAA) or relatively similar (MotifModeler) scores for the random genes compared to the pro-inflammatory genes (Table 2) stressing the specificity of the four motifs (Figure 4B, and Tables 1 &2) for regulating pro-inflammatory gene expression.

**Table 1:** Comparison of TFBS prediction scores between test *vs*. random gene sets.

Matrix Accession Number	TF name	MEME	MEME	Motif Modeler	Motif Modeler	PASTAA	PASTAA
		
		E-value*	E-value**	TCS score*	TCS score**	P-value*	P-value**
M01014	SOX	1.60E+07	-	1.49E-02	7.06E-03	2.99E-01	7.38E-01

M00699	IRF-8	6.90E-01	-	1.50E-02	-	3.50E-05	9.51E-01

M00233	MEF-2	1.60E+07	-	1.54E-02	6.91E-03	5.75E-01	7.68E-01

M00062	IRF-1	2.60E-07	-	1.55E-02	6.86E-03	7.40E-05	-

* Score from the test gene set

** Score from random gene set

Blank cells represent TFBS that were not identified by the respective tool in the test or random set. For MotifModeler, higher the TCS score better the prediction whereas for PASTAA, lower the P-value better the prediction.

**Table 2:** Reciprocal comparison of TFBS prediction scores between random *vs*. test gene sets.

Matrix Accession Number	TF name	MEME	MEME	Motif Modeler	Motif Modeler	PASTAA	PASTAA
		
		E-value*	E-value**	TCS score*	TCS score**	P-value*	P-value**
M00649	MAZ	-	4.10E+05	1.73E-02	8.28E-03	2.49E-02	6.92E-03

M00423	Foxj2	-	1.80E+07	1.45E-02	7.25E-03	6.00E-01	5.05E-01

* Score from the test gene set

** Score from random gene set

Blank cells represent TFBS that were not identified by the respective tool in the test or random set. For MotifModeler, higher the TCS score better the prediction whereas for PASTAA, lower the P-value better the prediction.

In the antibacterial gene set, the tools found 22, 7, and 178 motifs respectively with the intersection containing only one motif (Prrx2 as the profile TF). Due to the limited size of this latter data set, we did not compare prediction scores for this data with a random data set, as we did for the pro-inflammatory data. However, we performed target identification for Prrx2 using literature sources (next section).

### Validation of TF-TFBS interactions controlling the pro-inflammatory genes

We approached the validation of the various predicted TFBS in the pro-inflammatory class primarily by (1) relating the profile TFs corresponding to the predicted motifs with experimentally identified target genes for those TFs, and then (2) checking the expression patterns of a subset of these transcriptional target genes in our microarray data (Bottom part of Figure 1). We manually characterized the target genes that various TFs act on by using the TRANSFAC database [15] and the Ingenuity Pathway Analysis tool [16]. Table 3 lists the pro-inflammatory profile TFs identified from the three TFBS prediction tools and their corresponding biologically validated target genes. A significant number of these genes have been implicated in the inflammatory response including Irf1, Irf2, Ifnb1, Tlr3, Stat1, Stat3, and Nfkb1 with a number of them regulated in LPS-stimulated macrophage activation [8]. To check for the expression patterns of these identified target genes within our data set, we filtered the microarray data by looking for two-fold induction in the N+L set compared to the N samples plus two-fold repression in the T+L samples compared to the N+L set. This filtering is similar to what is done earlier (Figure 1) except for the cut-offs being two-fold with no p-value threshold here; it resulted in an expression signature of 2283 genes corresponding to the pro-inflammatory phenotype.

**Table 3:** Biologically validated target genes of profile TFs predicted from the pro-inflammatory gene set.

Matrix Accession Number	TF	Target Genes
M01014	Sox5	Smad5, Smad1, Smad7, Sox6, **Sox5**, Mir125b2, Mir34a, Mir224, Mir15a, Mir125b1, Lipe, **Sry**

M01014	SRY	Slc9a3r2, Wt1, Akr1b10, Zfp748, Hdac3, Smad3, Ar, Importin beta, Ep300, Kpnb1, Kpna, Znf208, **Sry**, **Amh**, **Ptgds**

M00699	IRF-8	Spi1, **Irf1**, **Trim21**, Cops2, **Irf2**, Il12b, Il1b, Cybb, B2 m, Cbl, Irf4, Nfatc1, Ttraf6, **Stat1**, **Etv6**, beta2-mg, Cdkn2b, Il-12 p40, H-2Dd, H-2Kb, H-2Ld

M01014	Sox13	Smad7, Fgf3

M01014	Sox4	Mir199a1, Mir27b, Mir199a2, Mir206, Mir29c, Mir107, Mir34a, Mir95, Mir17, Mir199b, Mirn292, Mirn101b, Cebpa, Sdcbp, **Tcf4**

M01014	Sox2	Pou5f1, Pou2f1, Pax6, Lbx1, Pdx1, Meis1, Asc, Golga6, Nkx2-3, Otp, Dlx5, Otx1, Dlx4, **Isl1**, Zfhx3, Fbxo15, Fgf4, Hrc, Nanog, Spp1, Zscan10

M01014	Sox9	Ep300, Nr5a1, Kpnb1, Crebbp, Smad3, Smad2, Amh, Mia, Med12, Maf, Importin beta, Calmodulin, Ppargc1a, Ncadherin, Col2a1

M01014	Sox15	Fhl3, Pou5f1

M00233	Mef-2	Smarca4, Hdac4, Hdac9, Hdac7, Hdac5, Nfat, Mapk14, Thra, Ep300, Ckm, Myog, Mef2 d, Jun, Slc2a4, Srf, Mck

M00062	IRF-1	Agtr2, C2ta, **Nos2**, H-2kb, **Ptgs2**, **Tlr3**, H2-Dd, IL-12, IL-7R, **Stat1**, Ciita, **Nfkb1**, Rela, Stat5, Tap1, **Vcam1**, Psmb9, **Ifnb1**, **Stat3**, Irf8, Crebbp, Smarca4, Cybb

M01014	Sox6	Cenpk, **Sox5**, Dazap2, Hdac1, Ctnnb1, Pdx1, Ctbp2, Mir29a, Mir221, Mir222, Mir29c, Mir126, Ccnd1, Fgf3, Hbb-y

M01014	Sox18	Mef2c, **Vcam1**

**Figure 5:**
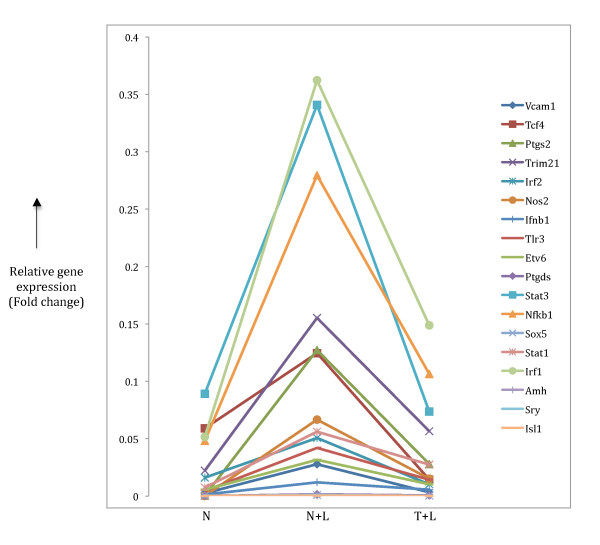
**Expression patterns pro-inflammatory target gene**. Target genes **highlighted **in Table 3 were checked to look for inflammatory gene expression pattern that is two-fold up regulation in N+L phenotype (compared to N) and further two-fold down regulation in T+L phenotype. The relative expression patterns of 18 target genes found in our microarray data are shown.

* Highlighted genes showed expression pattern as in Figure 5.

Profile TFs corresponding to the predicted motifs are searched against the TRANSFAC [14] and IPA [15] databases and the various target genes known to be associated with these profile TFs are listed. All profile TFs for each motif (Matrix Accession Number) in the databases are shown.

Interestingly, there were 18 genes from this larger pro-inflammatory gene set that overlapped with the target genes in Table 3 (**highlighted**). From the original pro-inflammatory gene set (of 228 genes), there were only four that overlapped with the target genes mainly due to the more stringent p-value filter that was imposed earlier. Next, we investigated the expression patterns of these 18 target genes in our microarray data. As expected, all 18 genes showed the pro-inflammatory phenotype (Figure 5) (p = 2.94 × 10^-4^). We also sampled 10 random sets of 18 genes each (generated using the RSAT tool [17]) for the pro-inflammatory pattern of expression in our microarray data (Additional file 2, Table S2) and found either no or very few genes (0, 1 or 2 out of 18) showing the pattern (p = 0.897). This target gene analysis clearly establishes our predictions as potentially genuine, biologically meaningful TF-TFBS interactions for the pro-inflammatory phenotype. A similar analysis on the two motifs (and the corresponding profile TFs, Maz and Foxj2, Table 2) from the original 228 random genes used in the TFBS prediction algorithms, identified a different set of 18 target genes (Additional file 3, Table S3) none of which had the pro-inflammatory expression pattern. As a further step to verify the role of these validated TF-TFBS interactions in establishing the pro-inflammatory phenotype upon LPS re-stimulation, we analyzed the expression levels of our 18 target genes in the Mages et al [10] data available in the public domain. Although these authors have added the tolerant (T) stage to their analysis (compared to the Foster *et al *data in our analysis), we could still look for gene expression patterns specific to the pro-inflammatory phenotype similar to our study. Essentially, we compared the fold change between the N and N+L stages [(N+L)/N] in their data with that between the T and T+L stages [(T+L)/T] with the former condition setting up the tolerant (T) phenotype and the latter showing differential gene expression of 'tolerizable' and 'non-tolerizable' genes. Almost all of our 18 transcriptional targets (17/18 with Isl1 being the exception) showed the pro-inflammatory expression pattern of being repressed (tolerizable) upon the second LPS treatment (Additional file 4, Table S4).

When we checked Prrx2, the only TF profile that was identified in our TFBS prediction from the antibacterial genes, it detected two genes (Ifi204, Pdgfra) as potential targets in the literature. Neither of these genes showed the antibacterial expression pattern in the Foster *et al *microarray data.

## Discussion

As a basis for characterizing transcriptomic signatures in LPS tolerance, we differentiated pro-inflammatory and antibacterial gene expression in LPS-stimulated macrophages. The genes in the Foster *et al *[6] microarray data were classified into pro-inflammatory and antibacterial sets using biologically relevant filters (Figures 1 and 2). Authors of that study had performed similar categorization to identify "tolerizable" and "non-tolerizable" classes corresponding to our pro-inflammatory and antibacterial classes respectively. Basically, Foster *et al *and we hypothesize that during tolerance although pro-inflammatory genes are down regulated, antibacterial genes need to be continually expressed. The two classes in their study were shown to have differences in chromatin modifications indicating transient silencing of some pro-inflammatory genes while priming of antibacterial gene expression [6]. However, they did not attempt to identify any global TF-TFBS interactions that can attest to transcriptional signatures responsible for the distinction between the two classes and hence to LPS tolerance. We have identified and characterized such distinctive interactions employing pattern search algorithms combined with literature-based validation of the target genes in the microarray data.

Our filtering approach deducted only a small number (18) of antibacterial genes since the cut-off of even one-fold step-wise increase from the N to N+L to T+L stages along with the p-value threshold seems to be highly stringent and this may indicate a highly specific antibacterial phenotype for these genes. Due to the small size of this data set, subsequent analyses of motif search, and target identification and validation for this class of genes did not merit much attention. However, genes such as Lcn2 [18] and Tirap [19] in this list (Table S1) have shown antimicrobial activity. Interestingly Lcn2 suppressed LPS-induced inflammatory cytokines in macrophages [20] indicating an additional anti-inflammatory role for this gene. It is likely that a number of antibacterial genes in our microarray data maintain the same level of expression at both the N+L and T+L stages to provide persistent bacterial protection and hence it would be worthwhile to analyze a larger set of these genes by filtering with a less stringent condition.

Based on the concept of coordinate gene expression being controlled by same or similar TFs binding to their cognate binding motifs, we identified respectively four and one TFBS motifs in the upstream regulatory regions of the pro-inflammatory and antibacterial genes (Figure 4). The essence of the specificity of these TF-TFBS interactions was provided by the intersection of the predictions from three tools of differing algorithmic approaches. Additionally, when compared to a random gene background the prediction scores for the four pro-inflammatory motifs were significantly higher in the test data set (Tables 1 &2). Our predictions identified interferon regulatory factors 1 and 8 (IRF-1 and IRF-8) as pro-inflammatory TFs (Figure 4, Table 1) and these proteins have been clearly implicated in macrophage-associated innate immunity [21-23].

We validated the TF-TFBS predictions in the pro-inflammatory class of genes by manually identifying the corresponding target genes implicated to be under control of the predicted TFs. This analysis produced a significant number of target genes and a small proportion of them (18 out of 141) showed the pro-inflammatory specific pattern of gene expression (Table 3, Figure 5). A similar gene expression pattern of the 18 genes identified in our analysis with data from the Mages et al [10] microarray analysis (Table S4) confirms the genuine pro-inflammatory phenotype of these transcriptional targets in macrophage tolerance induction, and indicates that the four predicted TFs most likely control the transcriptional regulation of these genes to establish the phenotype. A number of genes in this list have been experimentally associated with LPS-mediated macrophage activation including some important TFs such as IRF1, IRF2, Stat1, Stat3 and Nfkb1 [8] that are essential members of presumptive transcriptional regulatory networks. Nfkb1 has been identified as a major player in the downstream signalling pathways of LPS-stimulated macrophages [24] with a crucial role in the transcriptional regulation of a number of target genes [9,25]. More importantly, some of these target genes, such as Trim21, Ptgs2 and Nos2 (Figure 5) that show the sharpest drop in gene expression upon prolonged LPS treatment have been implicated in tolerance [26-28]. Nfkb1 acts as the controlling TF in LPS-induced expression of Ptgs2 and Nos2 [29] while acting downstream of Trim21 [26]. Here, since the microarray data represents a static view of gene expression, we cannot determine whether the Nfkb1 gene acts up or downstream of these genes as they all (Nfkb1, Trim21, Ptgs2 and Nos2) show the prototypical pro-inflammatory phenotype of down regulation (Figure 5) in induction of tolerance. A caveat to this classification may be the case of Nos2 that shows up in the pro-inflammatory category in our analysis. This gene has a direct role in killing intracellular pathogens [30-32] even though it shows down regulation in the tolerant state. A reason for the presence of such genes in our classification maybe due to the non-linearity of TF-TFBS interactions in that the dynamic modulation of transcriptional target gene expression does not always correlate with the corresponding TF binding to its site.

A limitation to this study is the lack of the tolerant (T) stage in the Foster et al [6] data set unlike the Mages et al [10] data. By employing the N+L stage instead of T (Figures 1, 2 and 5) we are likely to miss some genes (false negatives) that are tolerizable. However, the filtering approach that we employed is logically sound and did result in a significant number (228) of pro-inflammatory genes. Our motif prediction tools converged on 4 TF binding motifs that could co-ordinately regulate these genes (Figure 4). From a biological perspective, the 18 genes that are validated from the literature regards to being transcriptional targets of the four predicted profile TFs are most likely to be genuine candidates for establishing and/or maintaining the pro-inflammatory phenotype.

It is interesting to note that although the exhaustive work of Ramsey *et al *[8] looked at the dynamics of transcriptional programs in LPS-stimulated macrophages, they did not characterize the differential expression of the two categories of tolerant genes as belonging to the pro-inflammatory and antibacterial classes. We observed a number of common genes between our 228 pro-inflammatory set and their list of 1960 differentially expressed genes probably indicating a mixture of both classes of genes in their data. Litvak et al [9] have implicated one such gene, Cebpd, a TF, in a regulatory circuit discriminating between transient and persistent TLR4-stimulated signals. A search for the Cebpd binding motif in the regulatory regions of our two classes of genes (similar to their analysis) is likely to shed more light on the varying gene expression patterns in LPS tolerance induction.

## Conclusions

By combining distinct gene expression array data with motif scanning and literature-based biological validation, we have identified and characterized transcriptomic signatures categorizing pro-inflammatory and antibacterial classes of genes in LPS tolerant macrophages. We identified 228 pro-inflammatory and 18 antibacterial genes likely to be transcriptionally regulated by four and one TFs respectively. Further, employing literature resources, we observed a number of target genes corresponding to the predicted profile TFs in the pro-inflammatory set and a subset of these targets clearly showed the pro-inflammatory gene expression pattern corresponding to LPS tolerance.

## Methods

### Differentially expressed genes

The Foster *et al *microarray dataset (GSE7348) was downloaded from the NCBI-GEO database [33]*via *FTP protocol. This data set was derived from murine (C57BL/6 strain) bone marrow macrophages left untreated (N), stimulated with LPS for 24 hours and then re-stimulated for 4 hours (T+L) or treated only with the second stimulation for 4 hours (N+L). RNAs from these 3 conditions were hybridized to the Affymetrix Mouse Genome 430 2.0 arrays (in duplicates) to get raw gene expression values for 45101 probe sets [6]. Differentially expressed genes were selected based on their expression level differences in various experimental conditions. For gene selection, in addition to fold change, we also performed Student's two-tailed t-test by assuming heteroscedasticity between the naïve and tolerant macrophages to prove significant differences in expression between the two conditions. For a pro-inflammatory gene, the ratio of average gene expression values in N+L to N and T+L to N+L should be > one and < one respectively, and have the t-test p-value of < 0.05. On the other hand, for an antibacterial gene, the ratio of average gene expression values in both N+L to N and T+L to N+L should be > one, and have the t-test p-value of <0.05. In this study, we utilized random mouse genes as background and they were selected using the RSAT tool [17]. The Mages et al [10] data was downloaded from GEO (GSE8621) and appropriate differential gene expression filters (based on fold change) were applied to compare with the pro-inflammatory transcriptional targets identified in our analysis of the Foster *et al *data.

### Sequence retrieval

Regulator modules are generally located in the upstream and near the promoter regions of a gene. We considered this as our basis to retrieve the 1000 to +300 region, with respect to TSS, of all selected test and random genes. Gene sequences were retrieved from NCBI using a local tool [34] and repeats were masked using RepeatMasker [35].

### Identification of TFBS

We used three TFBS prediction tools *viz*., MEME [12], MotifModeler [13], and PASTAA [14]. We performed this analysis to both our test and random sets of genes. Description and parameters used for these tools are as follows:

#### MEME

It works by searching for repeated, ungapped sequence patterns in the input DNA with statistical significance. The non-default parameters used for this analysis comprised of number of motifs (30), maximum width (15), and mode of motif distribution (zero or one per sequence, zoops). Additionally the revcomp parameter was used to search for TFBS in both strands. MEME identifies motifs without any concern to biological validation.

#### MotifModeler

Gene UIDs and corresponding expression values of a co-regulated set of genes were given as input and this software works by taking a set of random motifs of fixed size and mapping them onto putative regulatory regions of genes of interest. A linear model was established by considering the expression values and the efficacy of selected motifs. In this model each motif was evaluated based on its contribution to transcriptional regulation. This was iterated many times to calculate a cumulative transcription contribution score (TCS) that was used for motif selection. Least square method was used to dictate inhibitory and stimulatory effects of the predicted motifs. For the analysis of random gene data, we used random expression values, generated by a PERL script, within the range of the maximum and minimum expression values of test genes.

#### PASTAA

This software takes ENSEMBL gene ids as input and ranks them based on the annotated binding affinities of 549 vertebrate TRANSFAC TFs to their promoter regions. Simultaneously, it also ranks them based on their tissue specificities derived from expression data. These two lists are compared for high ranked common genes and an iterated hypergeometric test is performed to deduce the relationships between TFs and the list of genes and corresponding p-values calculated. Lower the p-value better the prediction.

#### Motif selection and their corresponding profile TFs

Top 70% of the predicted motifs were selected and compared in all tools. MEME predicts the consensus sequences of all possible hits in the promoter region of a given set of genes but does not provide any annotated information about the predictions. Hence, we took the position weight matrices (PWM) of the predicted consensus sequences and searched in TRANSFAC [15] for the motif accession numbers using the TOMTOM tool [36]. MotifModeler and PASTAA provide motif accession numbers for the predicted motifs. We used TRANSFAC to manually map these motifs to TFs.

### Literature-based validation for target genes

We collected a list of genes (from TRANSFAC and IPA) that have been biologically identified as targets to the profile TFs that were discovered in motif prediction. We compared this list with the list of genes in the microarray that passed through a two-fold filter for the pro-inflammatory expression pattern. By normalizing the highest expression value in the N condition of the raw microarray data (45101 probe values) to 1, we calculated relative expression values for the filtered target genes corresponding to the N, N+L and T+L conditions (Figure 5).

## Competing interests

The authors declare that they have no competing interests.

## Authors' contributions

RKK participated in the conception of the study, designed and carried out all the experiments, analyzed the data and helped draft the manuscript. NBP conceived of the study, participated in its design and coordination and drafted the manuscript. Both authors read and approved the final manuscript.

## Acknowledgements

We thank M. H. Kaplan for critical review of the manuscript. RKK was supported by a research fellowship from the IUPUI Graduate Office.

## Supplementary Material

Additional file 1 **Table S1**. Click here for file

Additional file 2 **Table S2**. Click here for file

Additional file 3 **Table S3**. Click here for file

Additional file 4 **Table S4**. Click here for file
