# Supplementary material for: Motif prediction to distinguish LPS-stimulated pro-inflammatory vs. antibacterial macrophage genes
Source: Immunome Res. 2010 Sep 21;6:5. doi: 10.1186/1745-7580-6-5 (PMC2949756; doi:10.1186/1745-7580-6-5)
Supplement: Additional file 1 — Table S1. [file 1745-7580-6-5-S1.PDF]

Table S1: Pro-inflammatory and antibacterial genes in tolerant macrophages.

**Pro-inflammatory Genes**			**Antibacterial Genes**
1200016E24Rik			Acsl1
1600014C10Rik			Clec4e
1810063B07Rik			Ctsz
2210407C18Rik			Dnahc2
2310030G06Rik			Ercc8
4930420K17Rik			Gosr1
4933424B01Rik			Grina
5730508B09Rik			H2-T22
5830443L24Rik			H2-T9
9230105E10Rik			Lcn2
A230046K03Rik			Ms4a6d
A530023O14Rik			Nsl1
AA960436			Psmd11
Acacb			Saa3
Adora2b			Slamf9
Aebp2			Tirap
AI451557			Tspo
Angpt1			Ube2m
Ankrd17			
Anxa7			
Arhgef4			
Arid5a			
Arid5b			
Arl4a			
Armc8			
Arpc1b			
Atp10a			
Atp1a1			
Azi2			
B3gnt2			
Bcl3			
Bmp2k			
Cadm2			
Car2			
Ccdc6			
Ccdc86			
Ccl3			
Ccl4			
Ccnd2			
Ccnl1			
Cd47			
Cebpd			
Chd9			
Chic2			
Cmpk1			

Cox15			
Csf1			
D1Ertd622e			
Daxx			
Dcp2			
Ddx58			
Dedd			
Dyrk2			
E330016A19Rik			
Ehd1			
Eif2ak2			
Ep400			
Epb4.1l4b			
Evl			
Fam162b			
Fam40b			
Fam82a2			
Fbxo42			
Fbxw11			
Fez2			
Fnbp4			
Fndc3a			
Fndc7			
Foxp1			
Gbp6			
Gch1			
Gda			
Gfi1			
Glipr2			
Gm13007			
Gm5637			
Gm7582			
Gm8203			
Gm8833			
Gmppb			
Gna13			
Gnaq			
Golga3			
H2afz			
Hbegf			
Hhex			
Hk1			
Homer1			
Ifi203			
Ifi35			
Ifih1			
Ifit1			
Ifit3			
Igtp			

Il13ra1			
Il1a			
Il4ra			
Itga5			
Itgav			
Itpkb			
Jak2			
Jarid2			
Jdp2			
Kcnn4			
Khdc1b			
Kif12			
Kremen1			
Lcp1			
Lin7b			
LOC432459			
Lrch1			
Ltbp1			
Lysmd2			
Maml1			
Map3k7ip2			
Mapk1ip1l			
Mapkbp1			
March5			
Marcksl1			
Mfsd9			
Mitd1			
Mitf			
Mtmr14			
Mtmr7			
Mtus1			
Mx1			
Mx2			
Mycbp2			
Myd88			
Myst3			
NC_000067.5:37592977-37501401			
NC_000068.6:101637862-101555269			
Ncoa3			
Ncoa7			
Nek6			
Neo1			
Nfix			
Nfkbib			
Nfkbiz			
Nfxl1			
NT_165754.2:270408-297524			
Nt5c3			
Nup98			

NW_001032204.1:1395-1565			
Pcgf5			
Pde4b			
Pdzrn3			
Peli1			
Pftk1			
Pgs1			
Phc2			
Phip			
Phldb1			
Pigv			
Plekha2			
Plekhf2			
Plod2			
Pml			
Pnp1			
Pols			
Ppm1k			
Prkx			
Prpf38a			
Psd4			
Ptpn2			
Pyhin1			
Rap2c			
Rcan1			
Rffl			
Rin2			
Rnf125			
Rnf19b			
Rps6ka3			
S100a10			
Samhd1			
Sap30			
Sav1			
Senp1			
Sertad1			
Sf3b3			
Slc39a14			
Slc45a3			
Slc4a7			
Slco3a1			
Smarce1			
Snx10			
Socs1			
Socs3			
Socs7			
Spred1			
Spsb1			
St7			

Stat3			
Stat5a			
Tagap			
Tcf4			
Tcp10b			
Tcp10c			
Tiparp			
Tjp2			
Tle3			
Tlk2			
Tmem2			
Tnfrsf1a			
Tor1aip2			
Tor3a			
Tpbg			
Tpst1			
Trim12			
Trim15			
Trim21			
Trim25			
Trim26			
Ttr			
Ube2d3			
Uhmk1			
Usp18			
Usp42			
Vcpip1			
Whsc1l1			
Ythdf1			
Zcchc6			
Zdhhc21			
Zeb1			
Zfp273			
Zfp36			
Zfp710			
Zkscan6			
