# Supplementary material for: Motif prediction to distinguish LPS-stimulated pro-inflammatory vs. antibacterial macrophage genes
Source: Immunome Res. 2010 Sep 21;6:5. doi: 10.1186/1745-7580-6-5 (PMC2949756; doi:10.1186/1745-7580-6-5)
Supplement: Additional file 2 — Table S2. [file 1745-7580-6-5-S2.PDF]

Table S2: Pro-inflammatory genes in microarray among random sets of genes.

**Sample 1**	**Sample 2**	**Sample 3**	**Sample 4**	**Sample 5**	**Sample 6**	**Sample 7**
Mospd4	Brwd2	Ahnak2	Rps28	Ankrd1	Ptprh	Drd1a
Il1r1	Tmem32	Klhl10	2410015M20Rik	Ctnnd1	Spata9	Mtmr2
Fgd4	Crym	Ggta1	Treh	Apoa1bp	Pttg1ip	Smad1
Drctnnb1a	Cntfr	2610110G12Rik	Scn3b	Avp	Mrpl30	Uox
Ephx2	Spp1	Plch2	Sptlc2	Gas2l2	Abcd1	Usp48
Htr1b	Psip1	Mgl1	Pcx	Atpbd3	Arhgap9	Zc3h4
2410018C20Rik	Rab21	E2f3	Ncor1	Slc6a8	2310002L09Rik	4933407I18Rik
4922501C03Rik	Slc29a1	Hist4h4	Rora	1700120K04Rik	Myh8	Cpxm2
Tspan32	Ptprn2	2700094K13Rik	Atad2b	Ttc30b	Ltc4s	Paxip1
Ndufa9	Fermt3	Sfpi1	Rbm3	Pik3cg	Smn1	Hnrpa3
Sema3g	1810015A11Rik	Hdgf	Nfxl1	Herc4	Tbc1d9	Tbx20
Klf7	Ly6h	2810455K09Rik	Rnasen	1700008J07Rik	Arhgap29	5930422O12Rik
Rab11fip1	Lman2l	5730494N06Rik	Ciao1	Dnajc14	Olfr1335	Ntan1
Stfa2l1	Tmem136	Ccnt2	Pbx3	Srgap3	Nudcd3	Tbkbp1
6330408A02Rik	Prdx3	2810025M15Rik	Ece2	Srms	Cxcr6	2610008E11Rik
Lrrc1	Rtcd1	Pax9	Gtf2h1	Bhlhb5	Armcx1	Htr2c
Wipi1	Dscr3	Spag4	Q9D061-2	Rpl13	Dtnb	Spc24
Gpx5	Serpini2	Fert2	Olfr979	Snx2	Olfr111	Nrp2

*Genes in red showed pro-inflammatory phenotype (being in the 2283 gene set)

	Pro-inflammatory	Target_genes	p-value
Random_sets	6	180	0.896997827
Whole_array	2283	45,101	

Sample 8	Sample 9	Sample 10
Macf1	Tcf20	9830130M13Rik
2810047C21Rik	A930005I04Rik	Copb1
Zfp780b	Mdfi	D830039M14Rik
Mipol1	Ccl21c	Hfe2
Ptprz1	Tgfbr1	Otoa
2300009A05Rik	Aqp1	Serpinh1
Prokr1	Fgf8	Mapt
Snph	TRGV6	Hdgf
Ppil5	1810010K12Rik	Armcx2
Suv39h1	Il3	4931428F04Rik
A2A8N3_MOUSE	Rhoj	1700022K14Rik
Rad51	Ckt2	4930455C21Rik
Kctd2	0610011F06Rik	Dirc2
Pkd1l3	Scg2	Htr2c
Ubap2	Immp2l	Thop1
Ngb	Gtf3c5	Ccdc120
9130409I23Rik	Ect2	Rnf125
Foxp1	Spg21	Rhoq
