# Supplementary material for: Motif prediction to distinguish LPS-stimulated pro-inflammatory vs. antibacterial macrophage genes
Source: Immunome Res. 2010 Sep 21;6:5. doi: 10.1186/1745-7580-6-5 (PMC2949756; doi:10.1186/1745-7580-6-5)
Supplement: Additional file 3 — Table S3. [file 1745-7580-6-5-S3.PDF]

Table S3: Biologically validated target genes of profile TFs predicted from the random gene set.

Matrix_Accession_Number	TF	Target_Genes
M00649	MAZ	Saa, Bptf, Cdkn1A, Sp1, Maz, Pnmt, Myc, Prkaca, Ins, Csnk2A1, Dcc, Mir133A1, Mir133A-1, Mir133A2, Sp4
M00423	Foxj2	Mir34A, Mir107, C4Bpa

Profile TFs corresponding to the predicted motifs are searched against the TRANSFAC [14] and IPA [15] databases and the various target genes known to be associated with these profile TFs are listed.
