# Supplementary material for: Motif prediction to distinguish LPS-stimulated pro-inflammatory vs. antibacterial macrophage genes
Source: Immunome Res. 2010 Sep 21;6:5. doi: 10.1186/1745-7580-6-5 (PMC2949756; doi:10.1186/1745-7580-6-5)
Supplement: Additional file 4 — Table S4. [file 1745-7580-6-5-S4.PDF]

Table S4: Validated pro-inflammatory genes in the Mages et al microarray.

ID_REF	Gene Symbol	Entrez Gene	FC_NvsN+L	FC_TvsT+L
1415989_at	Vcam1	22329	21.03534475	1.375125938
1434149_at	Tcf4	21413	0.940831861	0.383186907
1417263_at	Ptgs2	19225	71.21904114	10.2524598
1418077_at	Trim21	20821	3.33785924	1.203228815
1418265_s_at	Irf2	16363	0.759880714	0.580148398
1420393_at	Nos2	18126	2.370624752	1.210356855
1422305_at	Ifnb1	15977	2.552513128	1.269896743
1422781_at	Tlr3	142980	1.510292613	0.647202851
1423401_at	Etv6	14011	3.927878807	1.349468469
1423860_at	Ptgds	19215	1.300546448	1.023731588
1459961_a_at	Stat3	20848	3.654837242	0.791684696
1427705_a_at	Nfkb1	18033	3.488814678	2.19486416
1440827_x_at	Sox5	20678	0.982617252	0.922051965
1440481_at	Stat1	20846	5.149377835	1.882698563
1448436_a_at	Irf1	16362	3.809986101	0.981773616
1450573_at	Amh	11705	1.357799062	1.096892699
1450578_at	Sry	21674	1.359702938	1.108414239
1422720_at	Isl1	16392	1.055331179	1.213721414

FC_NvsN+L = Fold change of gene expression in the N+L stage compared to N [(N+L)/N]

FC_TvsT+L = Fold change of gene expression in the T+L stage compared to T [(T+L)/T]
